# The Dog Soundscape: Recurrence, Emotional Impact, Acoustics, and Implications for Dog Observations and Dog–Human Interactions

**DOI:** 10.3390/ani14020279

**Published:** 2024-01-16

**Authors:** Sophie Savel, Thierry Legou

**Affiliations:** 1 Aix-Marseille University, CNRS, Centrale Marseille, LMA UMR 7031, Marseille, France; 2 Aix Marseille University, CNRS, LPL UMR 7309, Aix-en-Provence, France; thierry.legou@univ-amu.fr

**Keywords:** dog, soundscape, acoustics analysis, sound frequency, bioacoustics, stress, emotional sensitivity, welfare, survey, audiometric methods

## Abstract

**Simple Summary:**

We proposed a descriptive model of the dog soundscape composed of 79 sounds classified into six categories. In a survey, 620 dog owners scored the recurrence of each sound, from never to daily, in their dog’s environment. The survey also revealed 25 sounds that are likely to elicit stress/fear, that is, negative emotional sensitivity, in dogs. The results indicate no beneficial effect of commonness and no deleterious effect of scarcity regarding sound events on sensitivity. For the sake of dog welfare, researchers, veterinarians, trainers, and owners may limit dogs’ exposure to the sensitive sounds identified in this study during their dog observations and dog–human interactions. A corpus of 84 sounds was spectrally analyzed. At the lowest sound frequencies, where canine hearing is poorest, negative emotional sensitivity was generally low. At the middle and high sound frequencies, sensitivity greatly varied across the sounds, which is incompatible with the general assumption. How emotional sensitivity relates to pitch and dog hearing sensitivity remains undetermined. We suggest that future behavioral audiometric studies may maximize the spectral spread of each sound while minimizing the spectral overlap between sounds to reduce both the testing duration and the risk of unintentionally targeting or missing frequency-dependent hearing impairments.

**Abstract:**

While numerous dog behavioral studies use environmental sounds, the dog soundscape remains undescribed. We proposed a list of 79 sounds classified into six categories: Dog, Dog accessories, Human, city and vehicles, Garden, countryside and weather, and Household. In a survey, 620 dog owners scored the frequency of their dog’s exposure to, and thus, the recurrence of, each of the 79 sounds, from never to daily. The survey results also extended to about 25 sounds the number of acknowledged sounds that are likely to elicit stress or fear, that is, negative emotional sensitivity, in dogs. Sound recurrence and emotional sensitivity were not correlated, showing no beneficial effect of frequent exposure to, and no deleterious effect of scarcity of, sound events. We suggest that for the sake of dog welfare, researchers, veterinarians, trainers, and owners may limit dogs’ exposure to the sensitive sounds identified in the study during their dog observations and dog–human interactions. A corpus of 84 sounds was collected. The sounds were spectrally analyzed by determining their F0 and 10 dB bandwidth parameters. At the lowest sound frequencies, where canine hearing is poorest, negative emotional sensitivity was generally low. At the middle and high sound center frequencies/F0s, sensitivity greatly varied from lowest to highest, which is incompatible with both the general assumption and dog auditory detection thresholds. How emotional sensitivity relates to F0 (pitch) and hearing sensitivity remains undetermined. Finally, we suggest that future behavioral audiometric studies of dogs may maximize the spectral spread of each sound while minimizing the spectral overlap between sounds so as to reduce both the testing duration and the risk of inadvertently targeting or, conversely, missing frequency-dependent hearing impairments.

## 1. Introduction

Over the last two decades, the number of behavioral studies of the domestic dog (*Canis familiaris*) has drastically increased, with interest in various topics, such as conspecific and interspecific communication, cognitive abilities, welfare, and, to a lesser extent, perceptual abilities—e.g., see [1] for a complete review. The studies among these that involve acoustic signals, either social or nonsocial, either received or produced by dogs, can be classified into three main categories.

The first category encompasses the numerous studies that aim to understand how dogs and their human caregivers perceive, categorize, interpret, and react to dog and human vocalizations in either conspecific or interspecific interaction contexts [2,3,4,5,6,7,8,9,10,11,12]. These studies assessed, for example, whether receivers are able to determine the identity, emotional state, or morphology of the signaler and the information conveyed by the signal. More objective studies examined the production mechanisms, classification, and acoustics parameters of canid vocalizations [13,14,15,16,17,18].

Studies in the second category revolve around negative emotional sensitivity to sounds in dogs, more precisely, around environmental sounds that are likely to elicit stress, fear, or anxiety in dogs. These studies investigate the different physiological (e.g., cortisol level, heart rate, etc.) and behavioral signs (vocalizations, body movements, and orientation) of emotional sensitivity in dogs to sounds, the possible relationship between sensitivity and acoustics parameters, the effects of sensitivity to sounds on cognitive abilities and welfare, the nature and efficiency of chemical and behavioral treatments, as well as the appropriateness of owner reactions [19,20,21,22,23,24,25,26,27,28,29,30,31,32,33,34,35]. With the exception of one study that involved several household sounds [26], the different studies cited focused on three sounds: thunderstorms, fireworks, and gunshots. It was established that the greater the level and the suddenness of a sound event, the greater the negative emotional sensitivity to this event. Very few studies mentioned the relationship between sensitivity and sound spectral parameters, and their results are not consensual. Although, it is often assumed that high-pitch and, to a lesser extent, low-pitch sounds, are more likely to elicit negative emotional sensitivity. Most studies relied on surveys completed by dog owners and revealed that the ability of owners to identify signs of stress, fear, or anxiety in their dogs depends on a variety of factors, such as educational level, gender, etc. As a result, the authors consider that both the number of sounds to which dogs are harmfully sensitive and the severity of this sensitivity are often underestimated by owners.

Studies in the third—and smallest—category are about the behavioral, non–physiological estimates of the hearing status (i.e., normal versus impaired) of dogs (see [36] for details) using physiological and behavioral audiometric methods in animals; see [37] for a comparative review of research on dog and human hearing. In past academic and clinical behavioral studies on the hearing status of dogs and cats, a few environmental sounds among the following were either directly produced or played back: hand clap, finger snap, dog vocalizations (bark, cry, yap, and whine), human pronouncing the dog’s name out loud, training clicker, squeaky toy, ceramic plate breaking, doorbell, whistling, vehicle siren, metal object shook or dropped on floor, and vacuum cleaner [38,39,40]. However, the authors made no mention of the recurrence (i.e., frequency of exposure), emotional valence (i.e., negative emotional sensitivity), and acoustics parameters (i.e., at-ear level, frequency region, spectral spread, etc.) of the sounds they used.

Dogs live in the environment of their human caregivers, so their soundscape shares a lot in common with that of humans. However, little is known about the degree of diverseness of the dog soundscape in terms of canine emotional sensitivity to and the acoustic characteristics of the different sounds that constitute this soundscape.

The first aim of the present study was to propose a descriptive model of the dog soundscape composed of 79 sounds classified into six source categories. The relevance of this descriptive model was assessed in a survey of 620 dog owners who scored the frequency of their dogs’ exposure to—thus, the recurrence of—each of the 79 sounds, using a scale ranging from never to daily.

A second aim of the study was to extend the list of acknowledged environmental sounds that are likely to elicit stress or fear, that is, negative emotional sensitivity, in dogs, in order to recommend their exclusion from academic and clinical dog observations and dog–human interactions for the sake of dog welfare. Therefore, we asked the 620 owners to report which sounds (either part of the descriptive model or not) they considered stressful/fearful for their dogs. We then examined whether negative emotional sensitivity was correlated with sound recurrence.

A third aim was to determine whether the negative emotional sensitivity of dogs to sounds was somehow correlated with objective acoustics characteristics. Therefore, we collected a corpus of 84 sounds from an online sound library and performed a spectral analysis of each sound (F0; 10 dB bandwidth). We then assessed whether negative emotional sensitivity to sounds was correlated with spectral characteristics. The sound level was not assessed because objective determination of “at-ear“ levels requires in situ measurements. At-ear sound levels, indeed, strongly depend on a variety of factors, such as, for example, the distance between the source and the receiver, the presence of absorbing or reverberating material, etc.

A fourth aim of this study was to examine the extent to which the use of a small set of environmental sounds is relevant in behavioral (academic or veterinary) examinations of dog hearing status. Precisely, we aimed to identify sounds that may not be eligible for audiometric screening according to the three following characteristics: negative emotional impact, spectral narrowness, and spectral redundancy between sounds. The last two characteristics are very important in behavioral audiometric screening regarding the very large bandwidth of the canine audible field [41].

## 2. Materials and Methods

### 2.1. Listing and Classification of the Various Sounds Constituting the Dog Soundscape

In the first stage of the study, we established a list of 79 sounds that are likely to constitute the soundscape of a dog living in either an urban or a countryside area. The list was based on a pilot survey of 120 dog owners and professionals (trainers, veterinarians, and breeders), who were asked to list all sounds they considered as “being part of the dog’s life”, as well as the cited literature above on dog hearing and behavior. Then, based on the standard classification of the human soundscape, the 79 sounds were classified into six source categories:

- Vocal and non-vocal sounds produced by dogs (e.g., bark, lapping water, etc.);
- Sounds produced by dog accessories (e.g., training clicker, squeaky toy, etc.);
- Vocal and non-vocal sounds produced by humans (e.g., word, laugh, scream, etc.);
- City and vehicle sounds (e.g., car passing, firework, etc.);
- Garden, countryside, and weather sounds (e.g., lawnmower, bird song, thunder, etc.);
- Household sounds (e.g., vacuum cleaner, dishes handled or breaking, door slamming or squeaking, etc.).

### 2.2. Dog Owner Survey of Sound Recurrence and Negative Emotional Sensitivity

#### 2.2.1. Survey Distribution

In the second stage of the study, a Google form addressed to dog owners was published online in both French and English (see Figure S1 in Supplementary Materials for a full copy of the English version). A French-American native speaker examined and confirmed the concordance between the two versions. Both versions were open from 20 June 2023 to 5 July 2023. A call for participation was published and shared on a public Facebook group of scientific popularization on canine hearing and deafness administered by the first author [42]. The call included a description of the survey and a direct link to the Google form.

#### 2.2.2. Survey Content

The sole inclusion criterion for participation was that the dogs owned by the participants were not deaf, considering that the study is about the “audible” dog soundscape. There were no other inclusion/exclusion criteria such as dog breed, age, gender, etc.

In the first question of the survey, owners were asked whether they were living in either an urban or a countryside area. In the following 79 questions, owners were asked to indicate “how frequently the sound in question was susceptible to be heard by their dog(s), regardless of whether this sound was soft or loud, close or far, pleasant or not, and elicited a reaction of the dog(s) or not”. Owners responded using the following five-degree scale, which allowed us to quantify the frequency of exposure to (referred to below as the “recurrence of”) each sound of the above-detailed list:

- 1 = “never”;
- 2 = “rarely”;
- 3 = “occasionally”;
- 4 = “frequently”;
- 5 = “daily”.

In the last open question of the survey, the participants were asked to report “which of the previously scored sounds they considered as stressful/fearful for their dog(s)”. Stress and fear, referred to below as “negative emotional sensitivity”, were not defined to the owners for two reasons. First, stress and fear are often confounded in past studies. Second, the behavioral signs of canine stress/fear, as well as their accurate identification by owners, are various—e.g., [23,26]. Although this was not specified, the participants were allowed to report sounds that were not listed in the survey.

Those participants who were living with several dogs were asked to complete the form only once. They were, however, allowed to include their different dogs when responding to the open question (e.g., “one of my dogs is scared/stressed by the vacuum cleaner, another by barks”, etc.). Participation was anonymous and took about 5 min.

#### 2.2.3. Participants and Ethics

In total, 620 dog owners completed the survey, including 573 for the French version and 47 for the English version. In agreement with most national demographics, 66% of the dog owners were living in a countryside area and 34% were living in an urban area. The participants gave their consent for the anonymous use of their responses in the study. The present study is part of a larger project that obtained approval from the Ethics Committee of Aix-Marseille University (number 003–07042022).

### 2.3. Collection and Analysis of the Sound Corpus

#### 2.3.1. Sound Collection and Settings

In the third stage of the study, we collected 84 sound samples in order to obtain at least one example of the 79 sound items listed in the survey, except for three, which we were unable to collect due to unsatisfying quality. In several cases, one sound item of the survey led to the collection of two sound samples. One sound was not listed in the survey but was included in the sound corpus because 12 participants in the survey had reported that their dogs were emotionally sensitive to this sound. All sounds except six (i.e., words pronounced by the first author) were obtained from a free online sound library [43]. The 84 sound files were set to the following parameters:

- .wav format;
- mono channel;
- 44.1 kHz sampling rate;
- 16-bit resolution;
- duration determined so as to render the sound event as “realistic” as possible.

#### 2.3.2. Acoustics Analysis of Spectral Parameters

If the acoustic signal is harmonic, it is possible to extract its fundamental frequency (F0, in Hz) as well as its other harmonic components (multiples of F0). F0 is the determining factor of the perceived pitch. F0 is frequently assessed in studies of vocalizations because it conveys information about both the inner state of the signaler and the communication message [44]. F0 is also used for soundscape descriptions [45]. In addition, it is frequently assumed that negative emotional sensitivity in dogs is greater for higher-pitch and, to a lesser extent, lower-pitch sounds. Therefore, we assessed the F0, averaged across the whole signal duration, of 60 of the 84 environmental sounds of the corpus. Thus, F0 was not extractable for 20 inharmonic sounds.

For all 84 sounds, we also assessed the lower and upper cut-off frequencies and the width and center frequency of the 10 dB bandwidth, a metric that has previously been used in bioacoustics studies—see, e.g., [46].

Each sound was sampled only once, but we are aware that the acoustics parameters analyzed are greatly sample-dependent. However, we verified that the spectral features, mainly the F0s, were consistent with the literature. The sound durations are solely reported below for informational purposes. These durations were fixed so as to obtain “realistic” samples of each sound event and, therefore, included either one long sound produced by a single source or three to seven sound bursts produced by one or multiple sources. As a result, the duration was not suitable for data analysis.

Adobe Audition software CS6 was used for signal processing (FFT with Hamming window, 2048 points). PRAAT software 6.1.08 was used for the extraction and visual verification of the F0. The magnitude spectra and the spectrograms of the 84 collected sounds, classified into six categories, can be seen in the Supplementary Materials (Figures S2–S7).

### 2.4. Data Analysis

#### 2.4.1. Recurrence Scores and Negative Emotional Sensitivity Reports

The mean “recurrence score” of each of the 79 sound items of the survey, comprised between 1 (never) and 5 (daily), was obtained by averaging across the 620 participants.

Sixteen participants gave either no or unclear responses regarding the sounds they considered as stressful/fearful for their dogs (e.g., “my dog is stressed by/scared of unpredictable sounds”, “…loud sounds”, etc.). Among the 595 remaining participants, 62 responded that their dogs were negatively sensitive to absolutely no sound. Four of them indicated that their dogs had been desensitized to sounds. The 542 remaining participants (i.e., 87% of the total cohort) reported at least one sound, either listed in the survey or not, to which their dogs were negatively sensitive. We assessed the percentage of reports of negative emotional sensitivity for each (listed and freely reported) sound across these 542 participants.

For the set of 79 sound items for which recurrence scores were obtained, the relationship between recurrence score and emotional sensitivity report was evaluated using the Spearman correlation coefficient.

#### 2.4.2. Relationship between Negative Emotional Sensitivity and Spectral Parameters

It is frequently assumed that dogs are more likely to exhibit negative emotional sensitivity to sounds that have very high and, conversely, very low pitch. Therefore, using Spearman correlation coefficients, we assessed the relationship between emotional sensitivity reports and two spectral parameters: F0 averaged across the whole signal duration (total of 60 sounds) and the center frequency of the 10 dB bandwidth (total of 84 sounds). R software 3.6.2 was used for statistical analysis. The two-tailed *p* values reported in the text and figures were adjusted using Holm’s correction for multiple comparisons [47].

#### 2.4.3. Relevance of Sounds Used in Audiometric Studies

The relevance of 15 environmental sounds that have been used in past academic or clinical investigations of dog/cat hearing status was examined according to the three following variables of the present study: emotional sensitivity report and the width and center frequency of the 10 dB bandwidth.

## 3. Results and Discussion

For the 84 sounds of the corpus classified into six categories, Table 1 lists the recurrence score averaged across the 620 participants, the percentage of sensitivity reports assessed among the 542 participants who provided clear responses, and the spectral parameters.

### 3.1. Sound Recurrence

**Figure 1:**
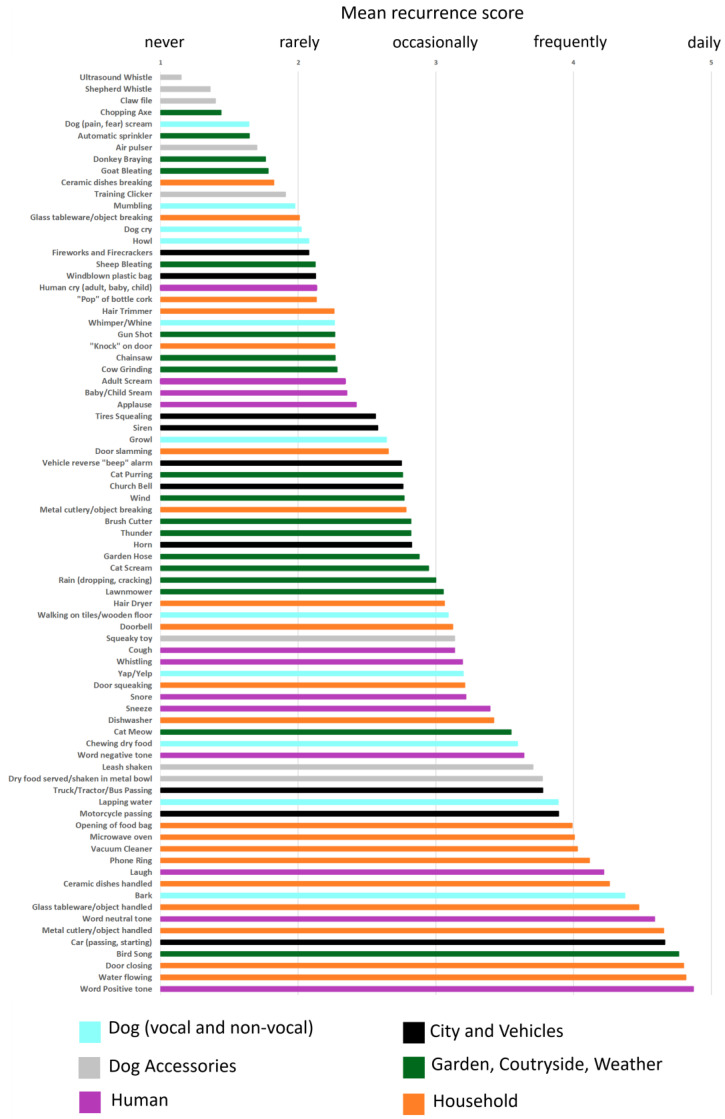
Mean recurrence scores averaged across the 620 participants for each sound item of the survey. Each bar color represents a different sound category (blue = “Dog”, grey = “Dog Accessories”, purple = “Human”, black = “City and Vehicles”, green = “Garden, Countryside, Weather”, and orange = “Household”). Because 95% confidence intervals amounted to only 0.1 for all 79 sounds, the error bars are not reported for clarity.

The recurrence scores averaged across the 620 participants are presented in Figure 1. Each category of the dog soundscape is denoted by a different bar color. Only three sounds, all in the “Dog Accessories” category (ultrasound whistle, shepherd whistle, and claw file) had recurrence scores close to 1 (“never”). A total of 85% (67 out of 79) of the sound items obtained recurrence scores greater than 2 (i.e., dog exposed more than “rarely”). Among the 36 sounds that had recurrence scores greater than 3 (i.e., dog exposed more than “occasionally”), all six categories are represented. Amongst the sounds that obtained the 20 highest recurrence scores, the most represented category is “Household”.

The goal of this analysis of owner-scored recurrence was to determine whether our first attempt to describe the dog soundscape in terms of both components and source categories was relevant. Based on the results, we conclude that at least 85% of the 79 sound items selected are realistic descriptors of the dog soundscape. The six source categories in which we classified the different sounds also seem relevant, provided that they all included several sounds that obtained substantially high recurrence scores. This first description of the dog soundscape, however depending on dog emotional sensitivity results presented below, could potentially be used so as to select auditory stimuli or markers in academic and clinical studies, as well as in dog-owner or dog-trainer interactions.

### 3.2. Negative Emotional Sensitivity

The reports of negative emotional sensitivity (in percentage) are listed for each sound in the third column of Table 1. Less than 22% of the 79 sound items of the survey were never reported as stressful/fearful. The top 10 of the sound items that obtained the highest emotional sensitivity reports are indicated by asterisks in the third column of Table 1. One of these ten sounds is in the “Dog” category: Bark (8.3%). However, the majority of the 45 owners who reported dog barks specified that these were stressful/fearful for their dogs only when the receiver dog was at home and the signaler dogs were out of view. Three sounds out of the top 10 are in the “City and Vehicles” category: Fireworks/Firecrackers (29.3%), Motorcycle (9.0%), and Truck/Tractor/Bus (7.7%). Three others are in the “Garden, Countryside, Weather” category: Thunder (39.3%), Hunting gunshot (18.5%), and Wind (9.0%). The remaining three sounds out of the top 10 are in the “Household” category: Vacuum cleaner (14.8%), Door slamming (12.7%), and Doorbell (10.1%). The “Dog Accessories” and “Human” categories are not represented in the top 10.

Sixteen other sounds were reported by more than 10 owners and, therefore, had sensitivity reports of 2.0% or more: Dog growl (3.1%), Air pulser (2.8%), Baby/Child scream (2.4%), Adult scream (3.1%), Car passing (4.1%), Horn (4.8%), Siren (2.2%), Rain cracking on surface (2.2%), Brush cutter (2.4%), Lawnmower (3.1%), Cat scream (3%), Hair dryer (4.8%), Ceramics breaking (3.1%), Metal object dropping (2.0%), “Pop” of bottle cork (2.0%), and “Knock-Knock” on door (2.0%). All six sound categories are represented. These sounds actually had low sensitivity reports (i.e., between 2% and 5%). However, it is well established that owners tend to underestimate or miss signs of emotional sensitivity to sounds in their dogs—e.g., [32].

Table 2 lists the sounds that were reported at least once as stressful/fearful but were not on the list of 79 sound items of the survey for which recurrence was assessed. The “Fighter jet” sound, reported by 12 owners, was added to the sound collection and analysis described below. The “fire/smoke and other alarms” sound in the “Household” category was reported but to a lesser extent than in a previous study [26]. Many sounds listed in Table 1 and Table 2 can be qualified as “sudden” (i.e., unpredictable), a variable that is frequently associated with negative emotional sensitivity to sounds in dogs.

Quantitatively, the present and past results on negative emotional sensitivity in dogs to sounds cannot be directly compared. We just asked our participants to report for a large variety of sounds which their dogs were sensitive to. Many past studies involved either one or a small set of sounds and asked their participants to score the degree or describe the behavioral signs of sensitivity in their dogs—e.g., [23,26,32].

Qualitatively, the present results are consistent with the literature on dogs’ negative emotional sensitivity to thunder, fireworks, gunshots [19,20,21,22,23,24,25,27,28,29,30,31,32,33,34,35], and household sounds [26]. However, the present study reveals additional, sometimes unexpected, sounds to which dogs are likely to exhibit negative emotional sensitivity. Many of these sounds are produced by sources that are typical of the human environment (“City and Vehicles” and “Household” categories). Thus, the main novelty of the present study is the large number of sounds, distributed between several source categories, that are likely to have a negative emotional impact in dogs. For some sounds, we suggest that emotional sensitivity may mostly arise from contextual aspects (e.g., “Doorbell” or “Knock-Knock on the door” are related to a stranger coming to the home).

In summary, we identified about 25 environmental sounds in the dog soundscape from various source categories that are likely to have a negative emotional impact in dogs. This list of “sensitive sounds” could be referred to by researchers, veterinarians, dog owners, and dog trainers (who use sounds in their dog observations or interactions) to ensure that such sounds have no deleterious effect on dog welfare. They could, thus, decide to either limit or avoid the dog’s exposure to certain sounds.

### 3.3. Relationship between Recurrence and Negative Emotional Sensitivity

**Figure 2:**
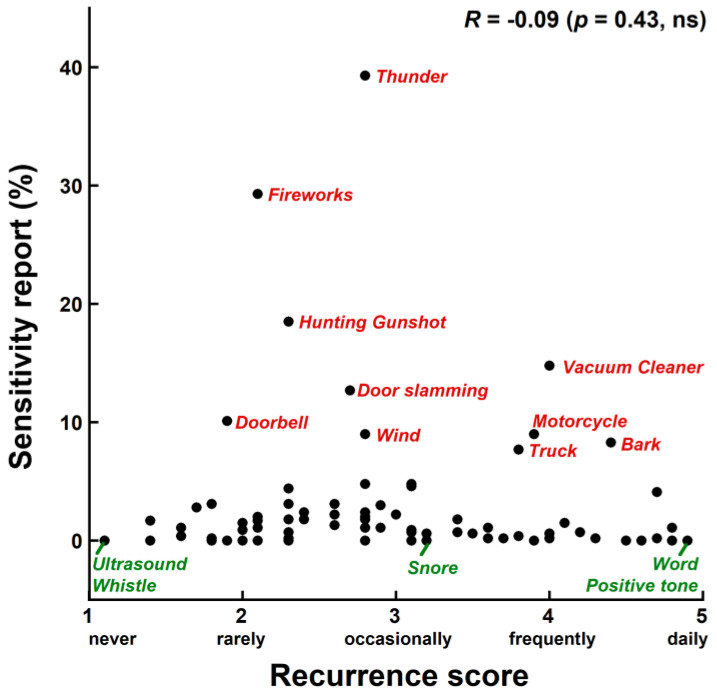
Mean recurrence score (ranging from 1 = never to 5 = daily) against reports of negative emotional sensitivity (in %) for the 79 sound items of the survey. The 10 sounds with the highest sensitivity reports, as well as three sounds with the lowest (conversely) sensitivity reports, are labeled in red and green, respectively.

Figure 2 presents reports of negative emotional sensitivity in percentage as a function of the recurrence score for the 79 sound items of the survey. We found no significant correlation between the two variables (Spearman’s *R* = −0.09; *p* = 0.43; *ns*). Sounds with the highest or, conversely, lowest emotional sensitivity reports had low, intermediate, or high recurrence scores indifferently. For the 10 sounds that were the most frequently reported to elicit negative emotional sensitivity, the recurrence scores greatly varied between 1.9 (“rarely”) and 4.4 (halfway between “frequently” and “daily”). In other words, dogs are not less emotionally sensitive to those sounds that they are the most frequently exposed to, and conversely, they are not more sensitive to the sounds that they are the least frequently exposed to. One implication of this is that frequent passive exposure to a sound may not have the beneficial effect of reducing the negative emotional sensitivity in the dog to this sound (see, e.g., vacuum cleaner, motorcycle, truck, and bark sounds in Figure 2). The opposite implication is that the scarcity of a sound event is not necessarily a reliable factor of negative emotional sensitivity. However, this does not exclude that *active* exposure with positive reinforcement can be very efficient in desensitizing a dog to a sound.

### 3.4. Acoustics Analysis

The different spectral parameter values (i.e., F0 averaged across the whole signal duration; lower, upper, and center frequencies, as well as width of the 10 dB bandwidth) are listed in Table 1 for each of the 84 sounds of the corpus. Twenty-four sounds (29%) had no extractable F0, among which were the three sounds that obtained the highest reports of negative emotional sensitivity (i.e., fireworks, thunder, and gunshots). Among the 60 remaining sounds, the F0 varied greatly from 60 Hz to about 10,000 Hz. Precisely, the F0 was less than 200 Hz for 12 sounds (14% of the corpus), between 200 and 500 Hz for 17 sounds (20%), between 500 and 1000 Hz for six sounds (7%), between 1000 and 4000 Hz for 19 sounds (23%), and between 4000 and 10,000 Hz for six sounds (7%).

Among the 84 sounds, the center frequency of the 10 dB bandwidth greatly varied from about 10 Hz to about 11,120 Hz. Precisely, the center frequency was less than 200 Hz for seven sounds (8% of the corpus), between 200 and 500 Hz for 20 sounds (24%), between 500 and 1000 Hz for 16 sounds (19%), between 1000 and 4000 Hz for 26 sounds (31%), and between 4000 and 11,120 Hz for 15 sounds (17%). In other words, the 500–1000 Hz and 1000–4000 Hz frequency areas were more represented when assessing the center frequency of the 10 dB bandwidth than when assessing the F0. This difference likely results from the 24 sounds with no extractable F0.

The median F0s and center frequencies, computed across the different sounds within each of the above-described frequency areas, were, however, similar (i.e., median F0 and center frequency = 115 Hz for the <200 Hz area, 350 Hz for the 200–500 Hz area, 770 Hz for the 500–1000 Hz area, 1500–1700 Hz for the 1000–4000 Hz area, and 5020–5490 Hz for the 4000–11,120 Hz area). This shows that the center frequency of the 10 dB bandwidth is a pertinent spectral metric that remains available even for inharmonic sounds with no F0.

These acoustics descriptive results reveal the diverseness of the dog soundscape, not solely in terms of recurrence and emotional sensitivity but also in terms of spectral parameters. This spectral variation implies that different sounds within the dog soundscape may have very dissimilar positions on the canine audible field.

### 3.5. Correlation between Negative Emotional Sensitivity and Spectral Parameters

**Figure 3:**
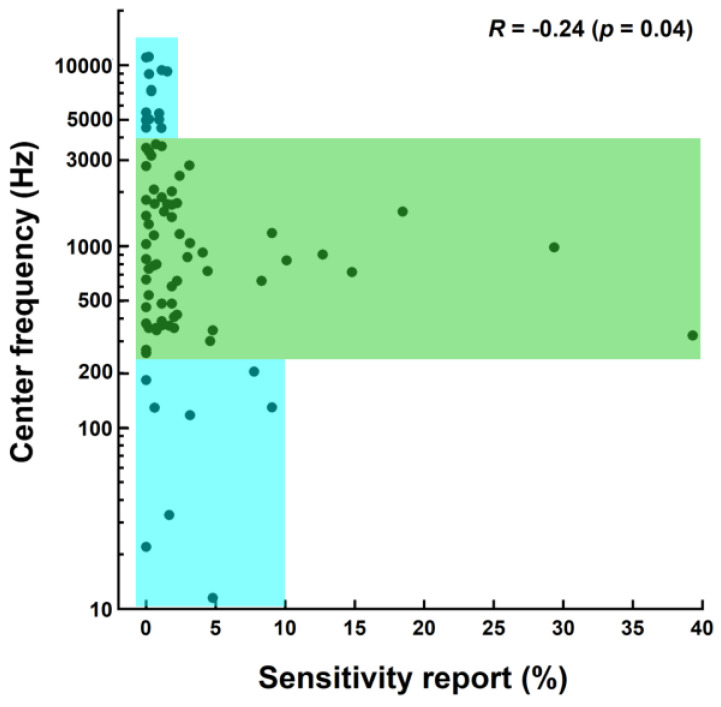
Report of negative emotional sensitivity (in %) against center frequency of the 10 dB bandwidth (in Hz, log scale) for the 84 sounds of the corpus. The blue areas encompass data points for which the sound center frequency is below 250 Hz or above 4000 Hz. The green area encompasses data points for which the sound center frequency ranges between 250 and 4000 Hz.

**Figure 4:**
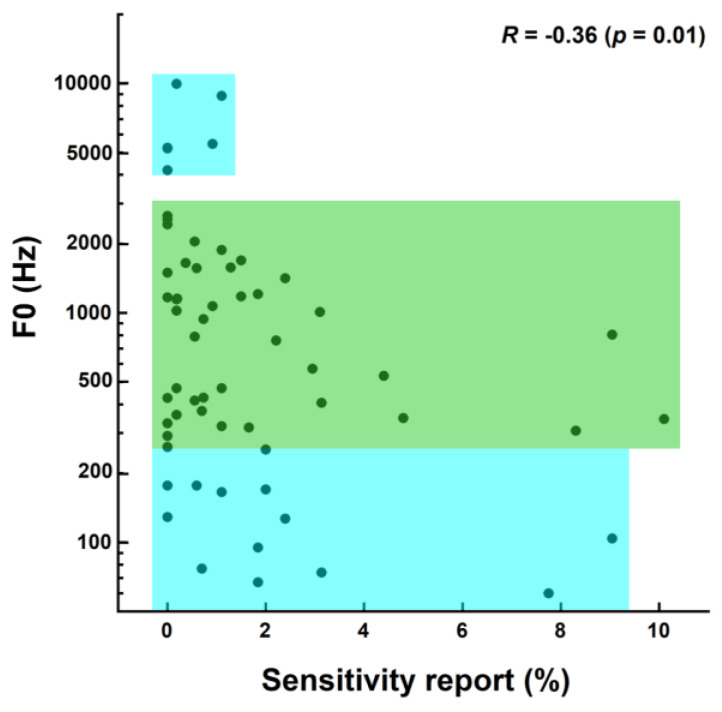
Same as Figure 3, except that the ordinate is for the F0, which we were able to extract for 60 out of the 84 sounds of the corpus.

Figure 3 presents the reports of negative emotional sensitivity as a function of the center frequency of the 10 dB bandwidth for the whole set of 84 sounds. There was a significant negative correlation between the two variables (Spearman’s *R* = −0.24; *p* = 0.04). Figure 4 shows the reports of negative emotional sensitivity as a function of F0 for the set of 60 sounds for which the F0 was extracted. Note that the five sounds with the highest sensitivity reports (see rightmost points in Figure 3) had no extractable F0, meaning that the x-axis upper limit is much smaller in Figure 4 than in Figure 3. A significant negative correlation was also found (Spearman’s *R* = −0.36; *p* = 0.01). The reports of emotional sensitivity were generally low for sounds with either the lowest (<250 Hz) or highest (>4000 Hz) F0s/center frequencies (see the lower and upper blue areas in Figure 3 and Figure 4) but showed a trend to decrease as frequency increased within the intermediary 250–4000 Hz area (see the green area in Figure 3 and Figure 4). This could partially account for the negative correlations reported.

The finding that negative emotional sensitivity was scarcely reported for the highest-frequency and lowest-frequency sounds is opposite to the common assumption that dog emotional sensitivity is greater (regardless of sound level and suddenness) for high-pitch sounds [26] and, to a lesser extent, low-pitch sounds.

**Figure 5:**
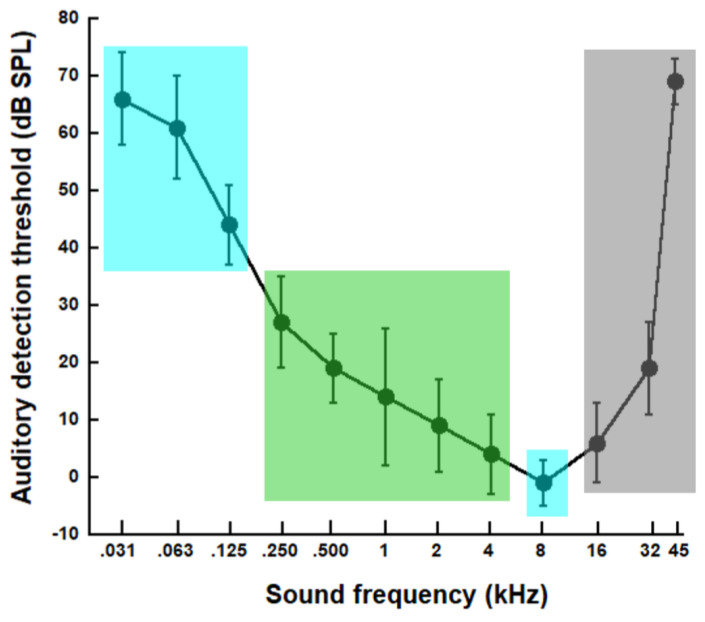
Canine auditory detection thresholds (in dB SPL) as a function of sound frequency (in kHz) averaged across four dogs in the study by Heffner [41]. The blue areas show the two regions of the center frequency and F0, for which we found low reports of negative emotional sensitivity. The green area encompasses the frequency region for which we observed a trend for emotional sensitivity to decrease as the center frequency or F0 increased. The grey area indicates sound frequencies that were not examined in the present study.

Below, we attempted to assess whether the present results related to the shape of the canine minimum audible field, which determines how hearing varies over the full frequency range of hearing. In the sole behavioral study of the canine audible field that we are aware of, detection thresholds were measured in four conditioned dogs in an anechoic room [41]. A tone, whose frequency varied between 31 and 45,000 Hz across trials, was played back. Figure 5 shows the “U” shape of the canine audible field thus obtained. Dog hearing is poorest at the lowest frequencies between 31 and 125 Hz, as well as at the highest frequency tested (45,000 Hz). As frequency increases from 125 to 8000 Hz, the detection thresholds decrease from about 45 to 0 dB SPL, thus reaching the frequency of best hearing. Then, as frequency increases from 8000 to 45,000 Hz, the detection thresholds increase from 0 to 70 dB SPL. The colored areas in Figure 5 allow us to compare this canine audible field to our results on negative emotional sensitivity. The blues areas indicate the two regions of F0/center frequency for which we found low negative emotional sensitivity: 10–200 Hz and 4000–11,000 Hz. The green area shows the [250–4000 Hz] frequency region for which we observed a trend for negative emotional sensitivity to decrease as the sound center frequency/F0 increased. The grey area represents the sound frequencies that were tested in [41] but are above those of our 84-sound corpus (maximum F0 in the corpus = 9988 Hz; maximum center frequency of the 10 dB bandwidth = 11,122 Hz; maximum upper frequency of the 10 dB bandwidth = 17,033 Hz; see Table 1).

At the lowest frequencies, where hearing is poorest, reports of negative emotional sensitivity were scarce. For the remaining frequencies of the audible field, however, the way emotional sensitivity reports varied with sound frequency is inconsistent with the auditory thresholds. As sound F0/center frequency increased from 125–250 Hz to 4000 Hz, negative emotional sensitivity reports decreased, while the auditory thresholds also decrease, meaning hearing *improve*. At sound F0s/center frequencies between 5000 and 10,000 Hz, the emotional sensitivity reports were scarce, and the hearing threshold is lowest (hearing sensitivity is best) at around 8000 Hz. This discrepancy between our emotional sensitivity results and the canine hearing thresholds, as measured in [41], is unexplained. One possible explanation is that environmental sound at-ear levels can be either close to or well above the canine detection threshold and, therefore, have either low or high canine loudness. In past studies of dog emotional sensitivity, sound spectral characteristics, precisely pitch, but also loudness, were described according to human perception [23,26]. There are, however, large differences between humans and dogs in the frequency range and threshold values of their respective audible fields. Another possible explanation is that the negative emotional sensitivity of dogs to sounds does not primarily rely on spectral characteristics but depends more on other acoustics parameters or even on non-acoustics factors.

### 3.6. Relevance of Environmental Sounds in Behavioral Audiometric Screening of Dogs and Cats

For 15 sounds used in canine/feline behavioral audiometric studies [38,39,40], Table 3 lists the percentage of negative emotional sensitivity reports and the width and center frequency of the 10 dB bandwidth. A total of 6 of the 15 sounds had sensitivity reports greater than 2%. Three of them were in the top 10 of the sounds most frequently reported as eliciting negative emotional sensitivity (see asterisks). We suggest that, in behavioral audiometric screening, such “sensitive” sounds should not be employed for obvious dog welfare purposes.

Regardless of negative emotional sensitivity, in Table 3, it can be seen that one of the three studies cited used seven different sounds to assess hearing sensitivity in dogs [38]. Many of their sounds had overlapping bandwidths and similar center frequencies (i.e., whine, bark, and yap sounds centered at about 600–800 Hz; hand clap and siren sounds centered at about 1700 Hz). Another of the three studies surveyed about 450 dog owners to determine what sounds veterinarians were using in clinical audiometric screening based on the dogs’ reactions to sounds [39]. Two of the six sounds reported in Table 3 (vacuum cleaner and doorbell) had similar center frequencies and overlapping bandwidths. The third study, which investigated the hearing status of 53 cats, used two different sounds (training clicker and squeaky toy) with almost identical bandwidths and center frequencies [40].

Thus, in future audiometric measurements using environmental sounds, we suggest that the spectral width of each sound should be as large as possible, and the spectral overlap between sounds should be as small as possible. By increasing the spectral width of each sound while minimizing the spectral overlap between sounds, a larger portion of the canine audible field would be covered. This would decrease the risk of involuntarily targeting or, conversely, missing frequency-dependent hearing impairments. Avoidance of spectral redundancy between sounds would have the additional advantage of reducing testing duration.

## 4. Limitations of the Study

The first limitation of the study is related to both the type of scale used for scoring sound recurrence and the data analysis performed on the scores. The dog owners were asked to indicate the recurrence of sounds using a five-degree Likert scale, with all degrees labeled: 1 = never, 2 = rarely, 3 = occasionally, 4 = frequently, and 5 = daily. The participants may all have understood that the scale is in increasing order, meaning that for degrees 2, 3, and 4, choosing 1 degree means that sound recurrence is greater than that for the preceding degree. However, some participants could possibly have encountered some difficulties in interpreting the “rarely”, “occasionally”, and “frequently” terms associated with degrees 2–4. One solution would have been to not label these three degrees or, alternatively, to have used a continuous slider scale with no graduation, ranging from never to daily. Moreover, we assessed the arithmetic mean score instead of the usual mode that is frequently assessed for ordinal scales. We did so because the mode only reflects the most chosen response, regardless of both the frequency of that choice and that of other response choices. Consequently, we noted that those sounds for which the recurrence modes were located on the extrema of the scale actually had a low frequency of responses (i.e., 25 to 39% of the 620 participants) and, therefore, had recurrence arithmetic means located *within* the scale. Therefore, we presented the arithmetic means so as to render a more realistic description of the distribution of the responses along the five-degree scale. The use of a slider scale, as evoked above, and arithmetic means would render even more precise estimates.

The second limitation of the study is that we investigated negative emotional sensitivity by asking owners to respond to an open question: “Which of the previously scored sounds are stressful/fearful for your dog(s)?”. It would be worth replicating the study by adding a few items to the initial list (see Table 2) and then asking owners to score the stressfulness/fearfulness of each sound using either a Likert or slider scale ranging from “my dog is NOT stressed/scared AT ALL by this sound” to “my dog is EXTREMELY stressed/scared by this sound”.

The third limitation relates to the sampling rate of the different sounds of the corpus. On the sound library from which they were extracted, the sounds had sampling rates of 44.1 kHz, and, therefore, the upper frequency of analysis was 22 kHz. This upper frequency is both usual and enough for human hearing studies. However, this 22 kHz upper limit is well below the frequency limit of 45 kHz of the canine audible field. It could be, therefore, argued that a sampling rate of 88.2 or 96 kHz, a rate that was rarely available on the sound library, would cover a larger part of the canine audible field. However, we assume that the 44.1 kHz sampling rate did not affect the spectral analysis presented because (1) the amplitude spectra of all sounds had very low relative amplitudes (100 dB below the maximum) at 22 kHz and (2) the F0s, center frequencies of the 10 dB bandwidth, and upper frequencies of the 10 dB bandwidth were well below 22 kHz for all 84 sounds. Thus, the rightmost part of the canine audible field may not be represented by the soundscape of dogs living with humans. In addition, dog hearing in this rightmost part of the canine audible field is quite poor. Note also that very few recording microphones, such as the ultrasonic microphones used, for example, in bats or dolphin bioacoustics studies, have good sensitivity above 22 kHz.

The fourth limitation relates to the uniqueness of the sounds of the corpus. Precisely, we collected a single sound sample for each sound component of the dog soundscape under study. The spectral metrics analyzed, that is, the F0 and center frequency of the 10 dB bandwidth, may slightly vary between different sound samples of a given sound type, such as, for example, between different signalers/emotional states of human or dog vocalizations or between different doorbells. However, these differences may not be large enough to affect the overall position of the sound on the canine audible field and, consequently, the present results.

## 5. Conclusions

The first aim of this study was to propose a first descriptive model of the dog soundscape. We selected 79 environmental sounds and classified them into six source categories. The relevance of this list was evaluated using a survey of 620 dog owners, who had to score the frequency of their dogs’ exposure to, thus, the recurrence of each sound, from never to daily. The results confirm the relevance of the six source categories and of at least 85% of the sound items.

The second aim of the study was to extend the list of acknowledged sounds (i.e., fireworks, thunderstorms, gunshots, and some household sounds) that are likely to elicit stress or fear, that is, negative emotional sensitivity, in dogs. The survey allowed for the identification of about 25 environmental sounds, distributed in different categories, that are likely to have a negative emotional impact in dogs. Most sounds can be qualified as sudden, a variable frequently associated with negative emotional sensitivity. No relationship between sound recurrence and emotional sensitivity was found. In other words, negative emotional sensitivity to environmental sounds in dogs may not decrease with regular passive exposure to, and, conversely, may not increase with scarcity of, sound events. Professionals (researchers, veterinarians, and trainers) and owners may choose to avoid or limit their dogs’ exposure to the sensitive sounds identified in this study to not affect dog welfare during their dog observations and dog–human interactions.

We collected a large sound corpus representative of the descriptive model of the dog soundscape. Spectral analysis (F0 and center frequency of the 10 dB bandwidth) revealed large spectral variations across the sounds. We found relatively scarce negative emotional sensitivity for the lowest sound frequencies, for which canine hearing is the poorest. Among the remaining part of the canine audible field, we observed an inconsistency between the way dog emotional sensitivity varied with sound frequency and dog auditory hearing thresholds. Overall, the results are not compatible with the general assumption that negative emotional valence in dogs is greater for high-frequency and, to a lesser extent, low-frequency sounds. We suggest that negative emotional sensitivity to sounds in dogs may rely on non-spectral or even non-acoustic factors. Further research is needed to identify these factors. We also suggest not relying on human-based descriptions of both the pitch and loudness of sounds, given the very large differences between human and canine hearing.

Finally, we assessed the relevance of environmental sounds in behavioral audiometric measurements of dog and cat hearing status. Previous studies used sounds for which we found substantial reports of negative emotional sensitivity in dogs. Moreover, we reported large degrees of redundancy between the different sounds of the same audiometric study (bandwidth and center frequency). We conclude that sounds with negative emotional impact may be excluded from behavioral audiometric testing for dog welfare purposes. Moreover, the spectral width of each sound may be as large as possible, and the spectral overlap between sounds as small as possible, so as to cover a larger portion of the canine audible field. This would reduce both the risk of unintentionally targeting or, conversely, missing frequency-dependent hearing impairments and testing duration.

## Figures and Tables

**Table 1:** Recurrence score (averaged across 620 participants who responded using a five-degree scale ranging from 1 = never to 5 = daily), report of negative emotional sensitivity (percentage of the 542 participants who clearly reported at least one sound, either listed in the survey or not, as being stressful/fearful for their dogs), and spectral parameters (F0, 10 dB bandwidth) of each sound classified into six categories. Sound sample durations are also reported, but only for informational purpose.

	Recurrence Score [1–5]	Sensitivity Report (%)	F0 (Hz)		10 dB Bandwidth (Hz)			Duration (ms)
				Lower F	Upper F	Width	Center F	
**DOG**								
Bark	4.4	8.3 *	307	345	947	603	646	242
Growl	2.6	3.1	74	47	188	141	117	5180
Yap/Yelp	3.2	0.6	788	754	818	64	786	85
Whimper/Whine ^(a)^	2.3	0.2						
*Whimper*			*1146*	*1292*	*1357*	*65*	*1325*	*339*
*Whine*			*360*	*366*	*711*	*345*	*539*	*1933*
Cry	2.0	0.9	5452	5211	5663	452	5437	299
Howl	2.1	1.7	317	280	452	172	366	6819
Mumbling	2.0	0.0	331	301	1766	1465	1034	2818
Scream ^(c)^	1.6	1.1	NA	NA	NA	NA	NA	NA
Lapping water	3.9	0.0	--	172	345	173	259	834
Chewing dry food	3.6	0.2	1024	1	711	710	356	4992
Walking on floor	3.1	0.0	5214	3122	6848	3726	4985	4004
**DOG ACCESSORIES**								
Leash shaken	3.7	0.2	9988	8376	13,867	5491	11,122	344
Dry food served/shaken in bowl ^(a)^	3.8	0.4						
*Dry food served in bowl*			*1650*	*1701*	*4651*	*2950*	*3176*	*819*
*Dry food shaken in bowl*			--	*1292*	*13,351*	*12,059*	*7322*	*156*
Training clicker	1.9	0.0	2556	1	9065	9064	4533	308
Squeaky toy	3.1	0.9	1071	1012	9022	8010	5017	544
Shepherd whistle	1.4	0.0	2647	1593	9388	7795	5491	812
Ultrasound whistle	1.1	0.0	--	11,003	11,047	44	11,025	339
Claw file (for grooming) ^(c)^	1.4	1.7	NA	NA	NA	NA	NA	NA
Air pulser (for grooming) ^(c)^	1.7	2.8	NA	NA	NA	NA	NA	NA
**HUMAN**								
Word with positive tone ^(a)^	4.9	0.0						
*“Yes” positive tone*			*2437*	*280*	*646*	*366*	*463*	*1127*
*“No” positive tone*			*261*	*215*	*711*	*496*	*463*	*949*
Word with neutral tone ^(a)^	4.6	0.0						
*“Yes” neutral tone*			*4192*	*108*	*646*	*538*	*377*	*501*
*“No” neutral tone*			*129*	*108*	*431*	*323*	*270*	*192*
Word with negative tone ^(a)^	3.6	1.1						
*“Yes” negative tone*			*8848*	*1*	*732*	*731*	*367*	*410*
*“No” negative tone*			*166*	*129*	*646*	*517*	*388*	*190*
Baby/Child scream	2.4	2.4	1418	1507	3402	1895	2455	1213
Adult scream	2.3	3.1	406	818	1270	452	1044	1229
Laugh	4.2	0.7	940	1	1593	1592	797	497
Snore	3.2	0.0	1171	86	280	194	183	2004
Cry ^(a)^	2.1	1.1						
*Baby cry*			*321*	*883*	*6266*	*5383*	*3575*	*809*
*Adult cry*			*471*	*431*	*538*	*107*	*485*	*2058*
Cough	3.1	*0.7*	428	237	452	215	345	712
Sneeze	3.4	*1.8*	1209	280	3747	3467	2014	1123
Whistling	3.2	0.6	2050	1593	2541	948	2067	871
Applause ^(a)^	2.4	1.8						
*One hand clap*			--	*409*	*2993*	*2584*	*1701*	*217*
*Crowd applauding*			--	*1*	*2907*	*2906*	*1454*	*5857*
**CITY AND VEHICLES**								
Car passing	4.7	4.1	--	65	1787	1722	926	4505
Motorcycle passing	3.9	9.0 *	104	108	151	43	130	5760
Truck/Tractor/Bus passing	3.8	7.7 *	60	43	366	323	205	6721
Horn	2.8	4.8	348	323	366	43	345	297
Siren (firemen, police)	2.6	2.2	759	775	2692	1917	1734	5066
Tires squeaking	2.6	1.3	1579	1421	1701	280	1561	1283
Vehicle reverse “beep” alarm	2.8	1.1	1883	1852	1895	43	1874	3081
Windblown/shaken plastic bag	2.1	1.7	--	1	65	64	33	4094
Fireworks/Firecrackers	2.1	29.3 *	--	172	1809	1637	991	5842
Church bell	2.8	1.8	95	43	926	883	485	9344
Fighter jet ^(b)^		2.2	--	43	797	754	420	9235
**GARDEN, COUNTRYSIDE, WEATHER**								
Brush cutter	2.8	2.4	127	108	2239	2131	1174	4309
Chainsaw	2.3	1.8	67	172	1034	862	603	9732
Lawnmower	3.1	4.6	--	86	517	431	302	6988
Automatic sprinkler	1.6	0.4	--	215	14,169	13,954	7192	8743
Garden hose	2.9	1.1	--	2 283	16,538	14,255	9411	5794
Axe chopping wood	1.4	0.0	--	43	1960	1616	851	2270
Hunting gunshot	2.3	18.5 *	--	65	3058	2993	1 562	1597
Cat scream	2.9	3.0	572	1	1744	1744	873	1049
Cat meow	3.5	0.6	415	409	1895	1486	1152	855
Cat purring	2.8	0.0	1500	1	43	42	22	4915
Bird song	4.8	0.0	5249	2 132	7773	5641	4953	1408
Cow grinding	2.3	0.0	177	172	1141	969	657	2673
Sheep bleating	2.1	0.0	426	517	3101	2584	1809	9244
Goat bleating	1.8	0.0	291	538	5017	4479	2778	585
Donkey braying	1.8	0.2	471	689	818	129	754	9443
Rain cracking on surface	3.0	2.2	--	65	1227	1162	646	7477
Thunder (clap)	2.8	39.3 *	--	86	560	474	323	7734
Wind (gust)	2.8	9.0 *	804	452	1916	1464	1184	9348
**HOUSEHOLD**								
Vacuum cleaner	4.0	14.8 *	--	108	1335	1227	722	4556
Hair dryer	3.1	4.8	--	1	22	21	12	6699
Ceramics dishes handled	4.3	0.2	1155	818	5836	5018	3327	5435
Ceramics dishes breaking	1.8	3.1	1011	1443	4156	2713	2800	799
Metal object handled	4.7	0.2	--	840	17,033	16,193	8937	1680
Metal object dropping	2.8	2.0	254	345	474	129	410	1759
Glass object handled	4.5	0.0	--	538	6481	5943	3510	2487
Glass object breaking	2.0	1.5	1181	1723	16,774	15,051	9249	767
Pop of bottle cork	2.1	2.0	170	215	495	280	355	270
Washing machine (dish, linen)	3.4	0.7	375	1	711	710	356	5345
Microwave oven	4.0	0.6	177	86	172	86	129	4867
Door closing	4.8	0.0	--	1	2950	2949	1476	234
Door squeaking	3.2	0.6	1567	1	3445	3444	1723	7405
Door slamming	2.7	12.7 *	--	43	1766	1723	905	773
Doorbell	1.9	10.1 *	345	818	861	43	840	1914
Knock-Knock on door	2.3	4.4	533	194	1270	1076	732	636
Phone ring	4.1	1.5	1696	1680	1744	64	1712	1179
Food bag opened	4.0	0.2	--	1	10,056	10,055	5029	1407
Hair trimmer (dog, human)	2.3	0.7	77	388	6934	6546	3661	5824
Water flowing	4.8	1.1	--	1	9001	9000	4501	2486

^(a)^ The different sound items signaled by ^(a)^ in the first column were assessed with a single question in the owner survey, but led to the collection of two different sound samples (see the sound names and values in italics). The six words were pronounced by a female speaker with a middle-pitch voice (first author). ^(b)^ The sound “Fighter jet” was not listed in the part of the survey assessing sound recurrence but was included in the sound corpus because 12 owners reported that their dogs were sensitive to (i.e., stressed/feared by) this sound. ^(c)^ The three sound items signaled by ^(c)^ in the first column were not spectrally analyzed because we were unable to collect sound samples of a satisfying quality (see “NA”, non available, in columns listing spectral parameters). * Values signaled by one asterisk in the third column are those that obtained the 10 highest percentages for the sensitivity reports. “--” symbols in the fourth column are for 24 sounds with no extractable F0.

**Table 2:** Sensitivity report, in %, for sounds that were reported at least once as being stressful/fearful for the dogs but that were not part of the 79 sound items for which recurrence was scored.

Sound	Sensitivity Report (%)
scream of unspecified species	3.7
unlisted or unspecified dropping items	2.8
unlisted (e.g., flying insect, chicken, horse, etc.) or unspecified animals	2.4
“human” scream	2.2
fighter jet	2.2
unspecified “blast”, “snap”, or “squeak” sounds	2.2
unlisted vehicles other than fighter jet (e.g., helicopter, hot-air balloon, etc.)	2.2
unlisted (i.e., hissing) or unspecified dog or cat sounds	1.7
music instruments (e.g., drums, violin, trumpet, etc.)	1.3
people talking outside	1.3
fire/smoke and other alarms	1.1
unlisted hand tools (e.g., electric drill, hammer, etc.)	1.1
bird scarer cannon	0.7
hydraulic brakes	0.7
foot steps	0.7
unlisted electric device (e.g., toothbrush, massage, fly screen, etc.)	0.7
unclassifiable	3.0

The “unclassifiable” row at the bottom is for 16 responses that we were unable to associate an event with the sound items listed in Table 1 and Table 2: “awning”, “bubbles in water dispenser”, “sport whistle”, “crumbled paper”, “flyswatter”, “folded plastic bottle”, “furniture moved”, “garbage container handled” (2 reports), “pétanque balls banging”, “shoot in football balloon” (2 reports), “umbrella opening”, “velcro scratch”, “wind turbine”, and “wood crackling”.

**Table 3:** Sensitivity reports and 10 dB bandwidth parameters (width and center frequency) for 15 sounds reported in three behavioral studies on canine and feline hearing status. For each study, the sounds are sorted in ascending order of center frequency.

Sound	Reference	Sensitivity	10 dB BANDWIDTH	(Hz)
		Reports (%)	Width	Center Freq.
Dog whining	[38]	0.2	345	539
Dog barking		8.3 *	603	646
Dog yapping		0.6	64	786
Hand clap		1.8	2584	1701
Vehicle siren		2.2	1917	1734
Ceramics plate breaking		3.1	2713	2800
Dog crying		0.9	452	5437
Metal object dropping	[39]	2.0	129	410
Vacuum cleaner		14.8 *	1227	722
Doorbell		10.1 *	43	840
Human adult yelling the dog’s name		3.1	43	1249
Whistling		0.6	948	2067
Metal object handled		0.2	16,193	8937
Training clicker	[40]	0.0	9064	4533
Squeaky toy		0.9	8010	5017

## Data Availability

The corpus of 84 sounds described in this study is available upon request to the corresponding author.

## Supplementary Materials

The following supporting information can be downloaded at https://www.mdpi.com/article/10.3390/ani14020279/s1, Figure S1: Copy of the survey; Figure S2: Magnitude spectrum (x-axis: frequency from 10 to 15,000 Hz; y-axis: relative amplitude from −30 to −75 dB; maximum relative amplitude of the signal set to −40 dB; dashed line = −10 dB cut-off) and spectrogram (x-axis: full signal duration; y-axis: frequency from 1 to 15,000 Hz) of the 11 sounds collected in the “Dog (vocal and non-vocal)” category; Figure S3: same as Figure S2 but for the 7 sounds collected in the “Dog Accessories” category; Figure S4: same as Figure S2 but for the 17 sounds collected in the “Human (vocal and non-vocal)” category; Figure S5: same as Figure S2 but for the 11 sounds collected in the “City and Vehicles” category; Figure S6: same as Figure S2 but for the 18 sounds collected in the “Garden, Countryside, Weather” category; Figure S7: same as Figure S2 but for the 20 sounds in for the “Household” category.
