# Supplementary figures and images for: The Dog Soundscape: Recurrence, Emotional Impact, Acoustics, and Implications for Dog Observations and Dog–Human Interactions
Source: Animals (Basel). 2024 Jan 16;14(2):279. doi: 10.3390/ani14020279 (PMC10812668; doi:10.3390/ani14020279)

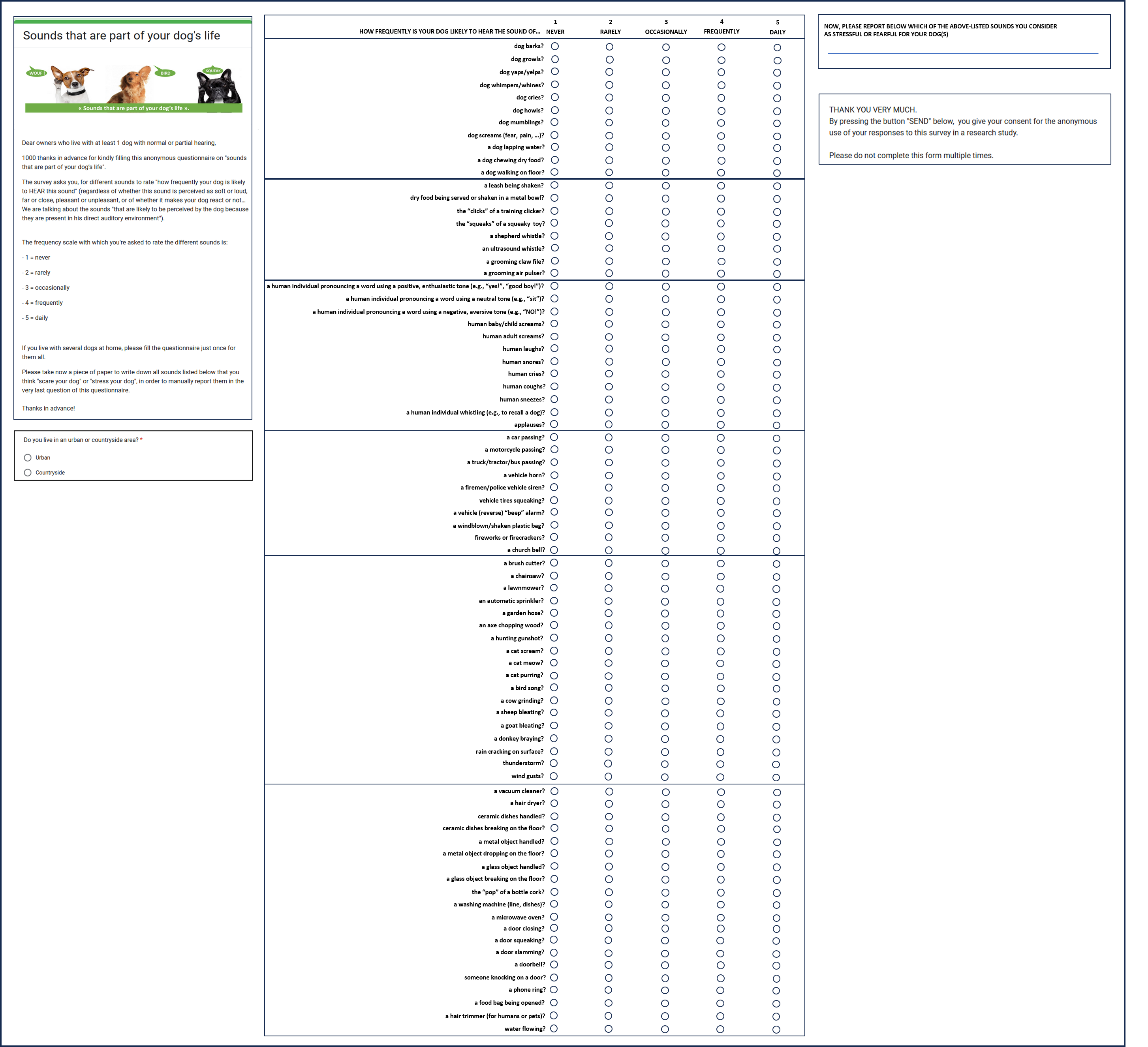

Supplement: Supplementary file 1 [file animals-14-00279-s001.zip › S1_FIGURE_SURVEY_COPY.png]

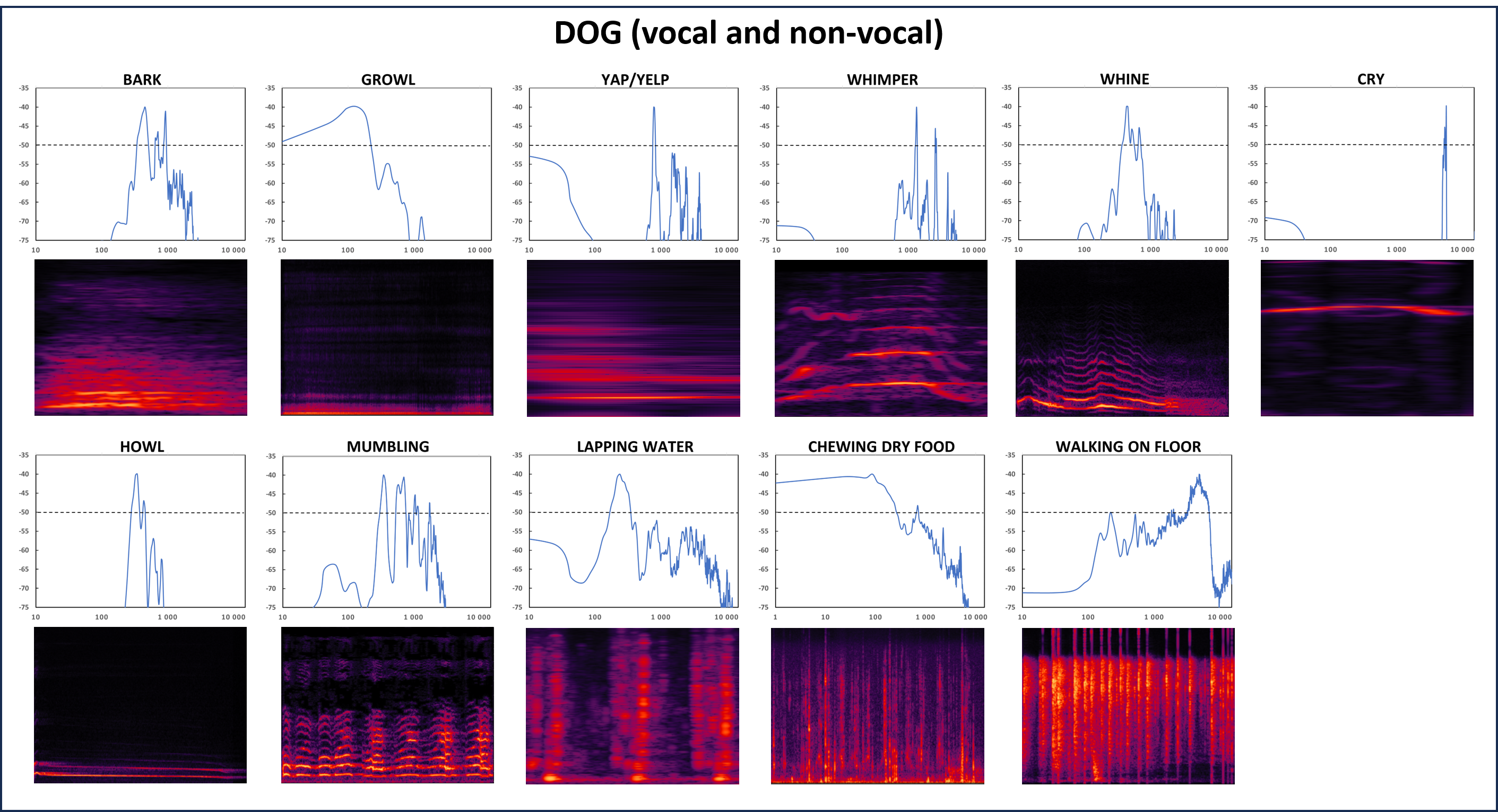

Supplement: Supplementary file 1 [file animals-14-00279-s001.zip › S2_FIGURE_SPECTRA_DOG.tif]

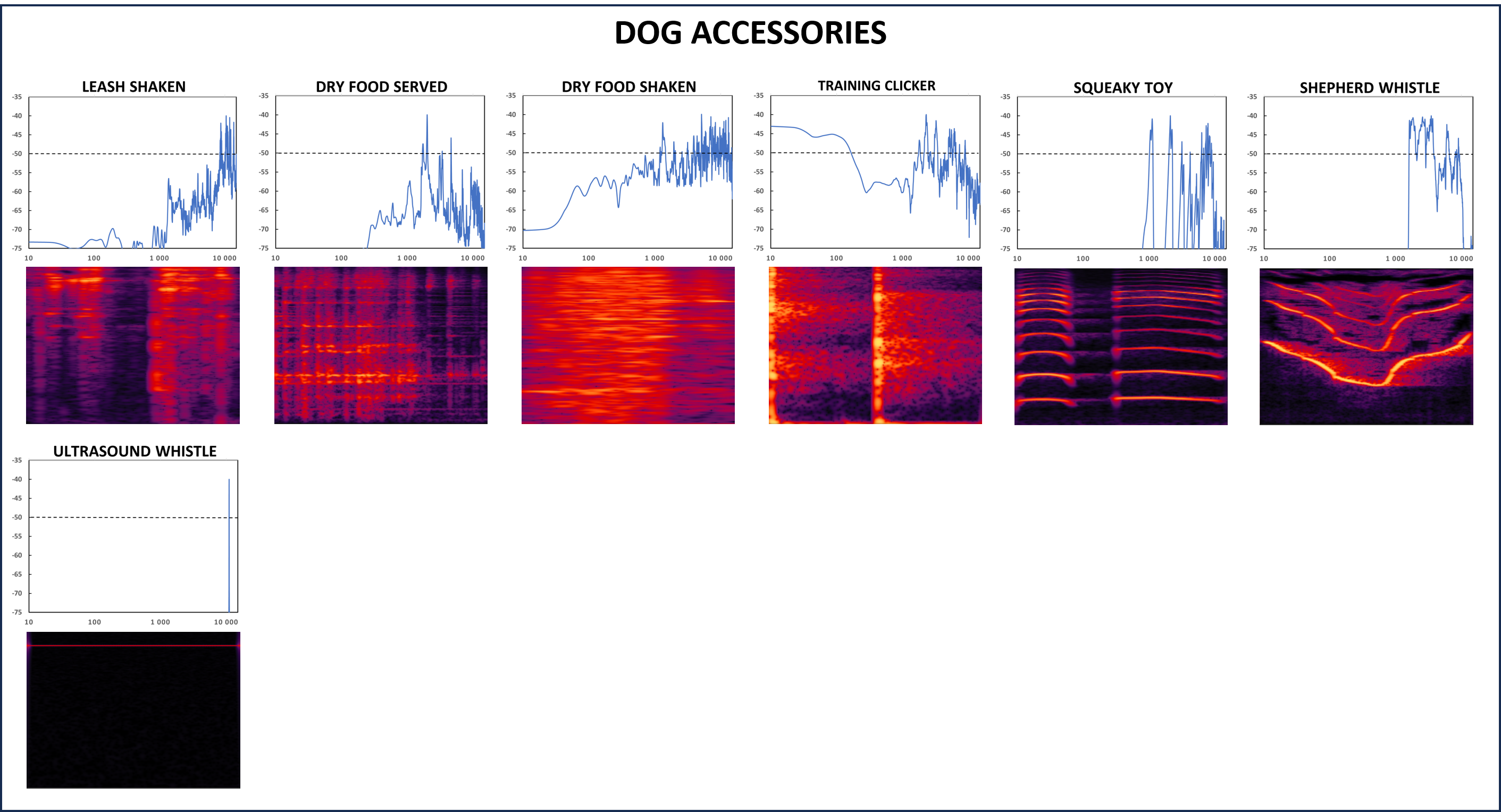

Supplement: Supplementary file 1 [file animals-14-00279-s001.zip › S3_FIGURE_SPECTRA_DOG_ACCESSORIES.tif]

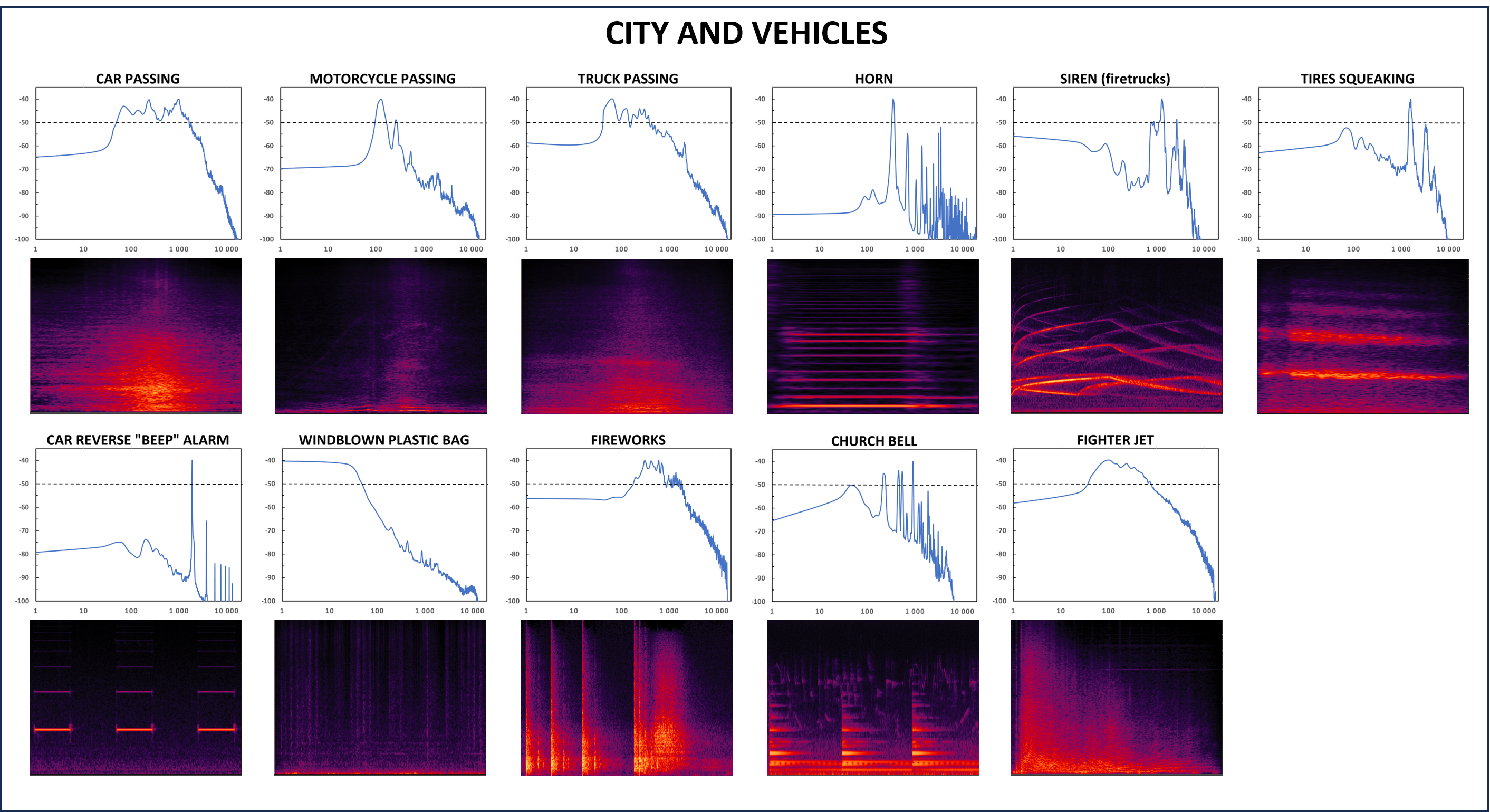

Supplement: Supplementary file 1 [file animals-14-00279-s001.zip › S4_FIGURE_SPECTRA_CITY_VEHICLES.tif]

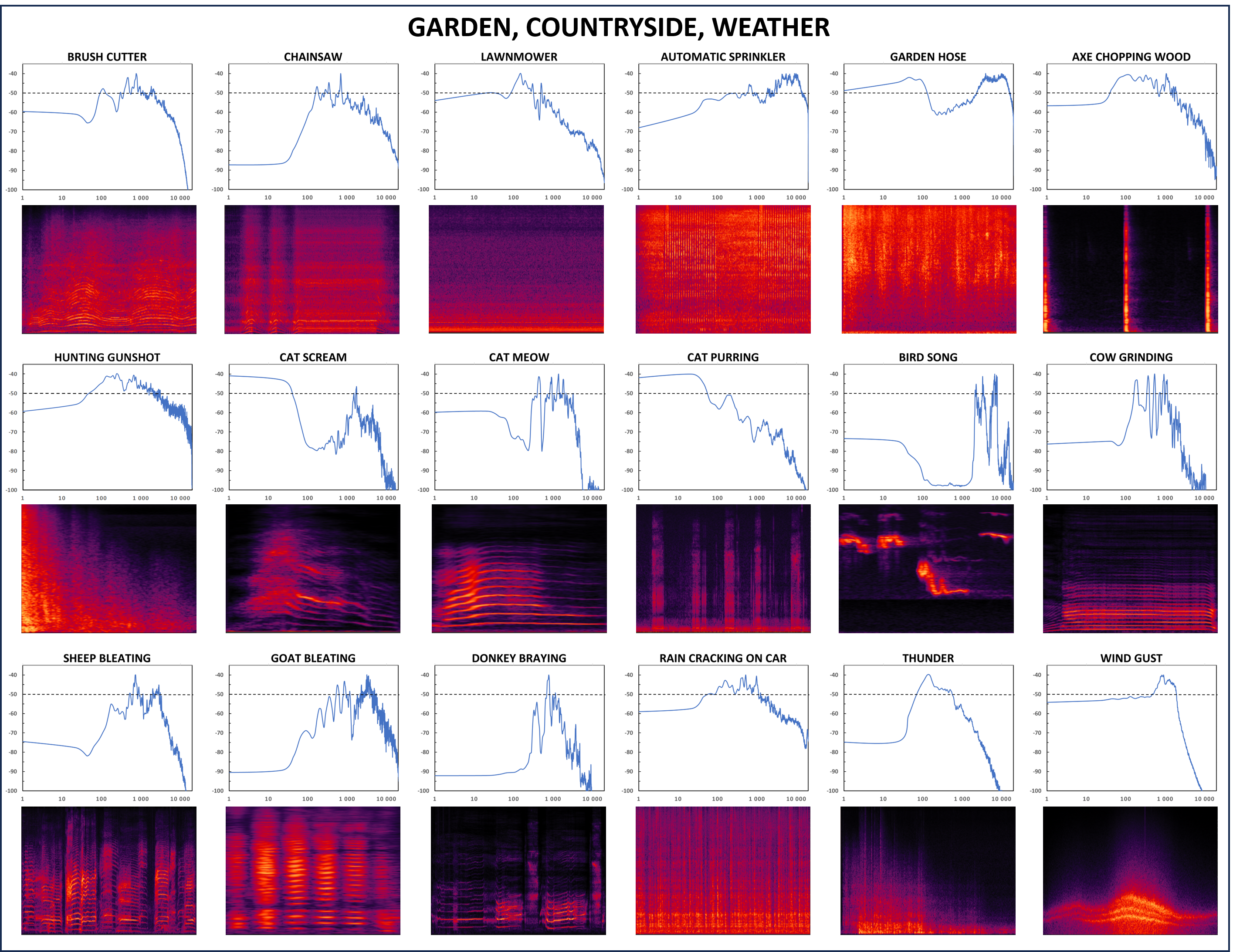

Supplement: Supplementary file 1 [file animals-14-00279-s001.zip › S5_FIGURE_SPECTRA_GARDEN_COUNTRYSIDE_WEATHER.tif]

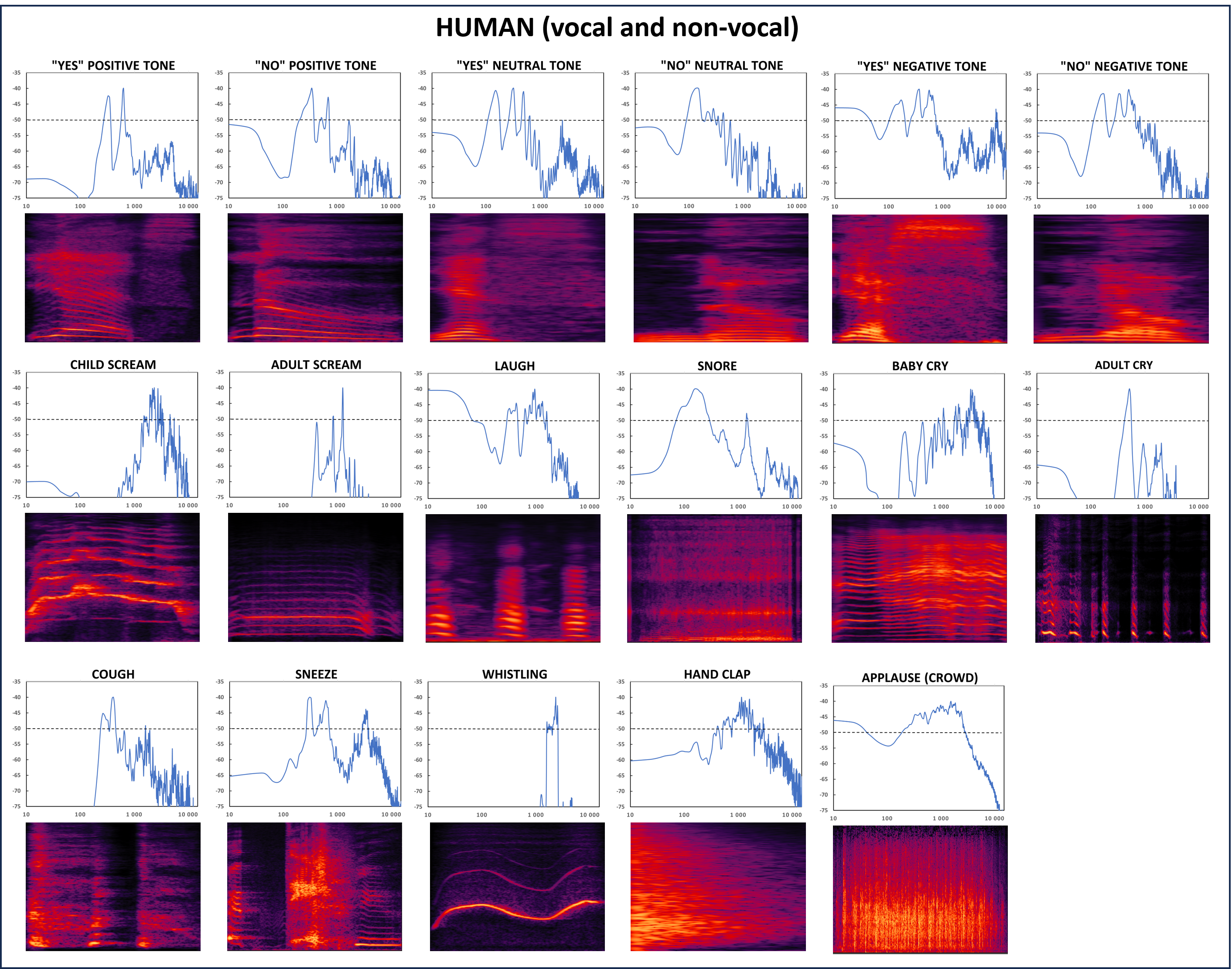

Supplement: Supplementary file 1 [file animals-14-00279-s001.zip › S6_FIGURE_SPECTRA_HUMAN.tif]

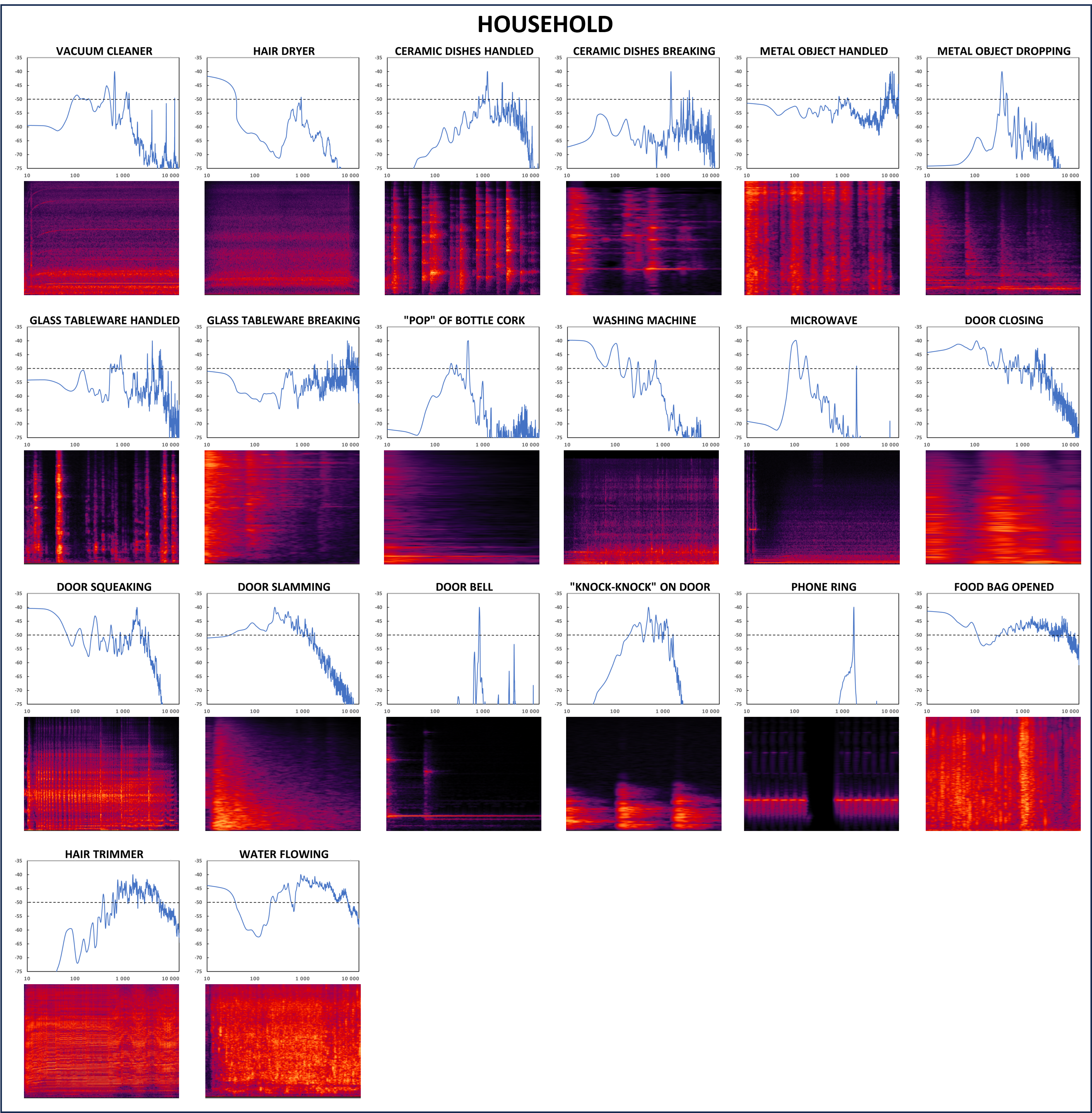

Supplement: Supplementary file 1 [file animals-14-00279-s001.zip › S7_FIGURE_SPECTRA_HOUSEHOLD.tif]
